# Understanding trophic interactions in host-parasite associations using stable isotopes of carbon and nitrogen

**DOI:** 10.1186/s13071-017-2030-y

**Published:** 2017-02-17

**Authors:** Milen Nachev, Maik A. Jochmann, Friederike Walter, J. Benjamin Wolbert, S. Marcel Schulte, Torsten C. Schmidt, Bernd Sures

**Affiliations:** 1 Aquatic Ecology, University of Duisburg-Essen, Universitätsstr. 5, D-45141 Essen, Germany; 2 Centre for Water and Environmental Research, University of Duisburg-Essen, Universitätsstr. 5, D-45141 Essen, Germany; 3 Instrumental Analytical Chemistry, University of Duisburg-Essen, Universitätsstr. 5, D-45141 Essen, Germany; 4 Department of Zoology, University of Johannesburg, PO Box 524, Auckland Park 2006, Johannesburg, South Africa

**Keywords:** Stable isotopes, Parasites, Trophic interactions, Acanthocephala, Nematoda

## Abstract

**Background:**

Stable isotope analysis of carbon and nitrogen can deliver insights into trophic interactions between organisms. While many studies on free-living organisms are available, the number of those focusing on trophic interactions between hosts and their associated parasites still remains scarce. In some cases information about taxa (e.g. acanthocephalans) is completely missing. Additionally, available data revealed different and occasionally contrasting patterns, depending on the parasite’s taxonomic position and its degree of development, which is most probably determined by its feeding strategy (absorption of nutrients through the tegument *versus* active feeding) and its localization in the host.

**Methods:**

Using stable isotope analysis of carbon and nitrogen we provided first data on the trophic position of an acanthocephalan species with respect to its fish host. Barbels (*Barbus barbus*) infected only with adult acanthocephalans *Pomphorhynchus laevis* as well as fish co-infected with the larval (L4) nematodes *Eustrongylides* sp. from host body cavity were investigated in order to determine the factors shaping host-parasite trophic interactions. Fish were collected in different seasons, to study also potential isotopic shifts over time, whereas barbels with single infection were obtained in summer and co-infected ones in autumn.

**Results:**

Acanthocephalans as absorptive feeders showed lower isotope discrimination values of *δ* ^15^N than the fish host. Results obtained for the acanthocephalans were in line with other parasitic taxa (e.g. cestodes), which exhibit a similar feeding strategy. We assumed that they feed mainly on metabolites, which were reprocessed by the host and are therefore isotopically lighter. In contrast, the nematodes were enriched in the heavier isotope *δ* ^15^N with respect to their host and the acanthocephalans, respectively. As active feeders they feed on tissues and blood in the body cavity of the host and thus showed isotope discrimination patterns resembling those of predators. We also observed seasonal differences in the isotope signatures of fish tissues and acanthocephalans, which were attributed to changes in food composition of the host and to seasonality in the transmission and development of acanthocephalans.

**Conclusions:**

This study provided first data on trophic interaction between an acanthocephalan species and its associated host, which support the tendency already described for other taxa with similar nutrition strategy (e.g. cestodes). Actively feeding taxa such as larval *Eustrongylides* sp., appear to act like predators as it can be seen from their isotope discrimination values. However, future research on additional host-parasite systems and especially on acanthocephalans is needed in order to corroborate these conclusions.

## Background

Parasites are increasingly considered as important components in ecosystems due to their diversity and biomass in which they occur under natural conditions [1, 2]. They can shape and modify food webs and can change their structure and stability in different ways [3–5] depending on the effects parasites have on their hosts. Accordingly, the direct interactions between parasites and their hosts are crucial for understanding parasite ecology. As a basis of these interactions, the trophic relations between parasites and their hosts have to be investigated [5]. Often, parasites are considered to act similar to predators [6], which is not justified from an ecological point of view, as they are characterized by much lower biomass in comparison to their hosts and usually only feed on one (host) organism [7]. Due to the fact that parasites exhibit different feeding strategies depending on their systematic relationships and their ontogenetic development, a generalization and overall classification concerning their trophic position in relation to their hosts is not possible. Generally, there are three ways of nutrient assimilation by parasites: (i) active feeding directly on host tissues, which could be considered as a predator-prey relationship; (ii) passive assimilation of products, that derive from the host’s metabolism; and (iii) sharing the same food source with the host, which represents a form of commensalism (e.g. [8]).

Stable isotope analyses can deliver insights into nutritional relationships between organisms. In ecology, the isotopic discrimination values of Δ*δ* ^13^C and Δδ^15^N are used as unique fingerprints, which allow for a determination of food sources and trophic interactions between organisms (reviewed in [9]). For example, the isotopic discrimination of carbon is used to differentiate food sources and that of nitrogen to determine trophic levels [10, 11]. Investigations of stable isotopes of nitrogen have shown that consumers are enriched in heavy nitrogen (Δ*δ* ^15^N) with an average of 3.4‰ per trophic level [12]. Accordingly, the application of stable isotope analyses might be helpful to elucidate the trophic relationship between a host and its associated parasite. So far, the number of studies based on stable isotope analyses of host-parasite interaction remains scarce. Although different parasitic taxa were considered so far (e.g. [13–15]) no information on acanthocephalans is available. Furthermore, the available data show contrasting patterns, with some endoparasites being depleted in *δ* ^15^N in comparison to their host tissues whereas others were enriched [5, 13, 14, 16–21]. However, often important factors such as developmental stage, feeding strategy and localisation of the parasite within the host were not considered, which are all crucial for the trophic interaction in host-parasite associations.

The aim of this study was to investigate the trophic relationships between the freshwater fish barbel (*Barbus barbus*) and its associated parasites using stable isotope analysis. For this purpose, barbels infected with either one parasite species (the intestinal acanthocephalan *Pomphorhynchus laevis*) or simultaneously with two parasites (*P. laevis* and larval nematodes of the genus *Eustrongylides* sp.) were collected from the River Danube in Bulgaria (see also [22]). The selected parasites belong to different systematic groups (Acanthocephala *versus* Nematoda) and inhabit different microhabitats in the host and were also found as different developmental stages. The barbel serves as definitive host for the acanthocephalan *P. laevis*, harboring the parasites in the intestine, where they mature and reproduce and live for 7–8 months [23] and references therein). Nematodes of the genus *Eustrongylides* exhibit a three-host life-cycle, with oligochaetes of the genus *Tubifex*, *Limnodrilus* and *Lumbriculus* as first intermediate hosts*.* Here, the parasites develop into third-stage larvae (L3), which are infectious for the second intermediate host, various planktivorous and benthivorous fishes. Barbel and other cyprinids can acquire infection, when feeding on infected oligochaetes. Within their fish host the nematodes molt into the fourth-stage larvae (L4) [24] and live in the abdominal cavity as freely migrating larvae or coiled under the thin serosa membrane [24]. The definitive hosts of the nematodes are fish-eating birds such as cormorants [24].

## Methods

### Fish sampling

The fish were collected from the River Danube in Bulgaria close to the town of Kozloduy at river kilometre 685 (for details regarding the sampling location see [25]). Sampling was conducted during summer and autumn of 2006. Ten fish caught in summer were infected only with the acanthocephalan *P. laevis* and eight fish sampled in autumn were infected simultaneously with the acanthocephalan *P. laevis* and the nematode (*Eustrongylides* sp.). Following capture, the fish were frozen (-20 °C) and transported into the laboratory, where the parasitological investigations and sample preparations were performed. After thawing, morphological parameters such as weight, total and standard length as well as body height were recorded. Subsequently, the Fulton’s condition factor of each fish (k) was calculated according to Schäperclaus [26] (k = 100 × W/L^3^). During fish dissection, the body cavity and the intestine were checked for parasites. All acanthocephalans were removed from the intestine, counted and frozen at -20 °C. Individuals of the nematode *Eustrongylides* sp. were collected from the body cavity, counted and also frozen at -20 °C until further processing. Samples of the fish tissues muscle, intestine and liver were taken and were kept frozen until stable isotope analyses. Parasitological parameters such as prevalence (P, %) and mean intensity (MI) were determined according to Bush et al. [27] and are summarized in Table 1.

**Table 1:** Morphological and parasitological parameters of barbel (*Barbus barbus*)

Parameter	Summer	Autumn
Weight (g)	555.5 ± 224.3	965.9 ± 445.8
Total length (cm)	38.2 ± 5.8	48.5 ± 4.8
Standard length (cm)	31.7 ± 4.9	40.0 ± 6.3
Body height (cm)	7.7 ± 1.1	9.3 ± 1.6
Condition factor	0.96 ± 0.09	0.82 ± 0.28
MI (range) - *P. laevis*	88.0 (37–197)	133.1 (31–307)
MI (range) - *Eustrongylides* sp.	–	22.8 (4–68)

*Abbreviation*: *MI* mean intensity

### Stable isotope analysis

Parasite and fish tissue (muscle without bones, intestine and liver) samples were freeze-dried using a freeze dryer (Heto PowerDry LL3000; Thermo Fisher Scientific, Waltham, USA) and subsequently powdered using a porcelain mortar. Standards and prepared samples were stored in a glass desiccator. A Sartorius SE2 analytical balance (Sartorius, Goettingen, Germany) was used to weigh all samples and standards. Samples (400–700 μg) were weighed into 4 × 6 mm tin foil capsules for solids (IVA Analysentechnik e.K., Meerbusch, Germany) by folding.

A MAT253 isotope ration mass spectrometer (IRMS) was connected *via* a ConFloIV interface (both Thermo Scientific, Bremen, Germany) to an EA1110 elemental analyzer (EA) (Carlo Erba Instruments, Milano, Italy). The EA was equipped with an AS200 auto sampler (also Carlo Erba Instruments). The operating software for the IRMS, the ConfloIV interface and the EA1110 was Isodat NT 3.0 (Thermo Scientific, Bremen, Germany). Helium of purity 5.0 (from AirLiquide, Oberhausen, Germany) was used as carrier gas for the EA-IRMS system. As reference gases nitrogen and carbon dioxide (both AirLiquide, Oberhausen, Germany) were used. Prior to measurement, the folded tin capsules were placed in the sample carousel of the autosampler from which the capsules were dropped one by one into the EA combustion tube. The quartz glass combustion tube (45 cm long, 1.8 mm o.d., 15 mm i.d.) was held at 900 °C and filled (from the bottom) with (i) ~0.4 cm quartz wool, (ii) ~0.5 cm silvered cobaltous/cobaltic oxide, (iii) ~0.4 cm glass wool, (iv) 10 cm copper oxide, and finally ~1 cm glass wool (all parts purchased from IVA Analysentechnik e.K., Meerbusch, Germany). Following capsule dropping, a pulse of oxygen (70 s) was introduced, which rises the temperature to ~1,700 °C, replaces the helium and combusts the sample immediately to CO_2_, H_2_O, O_2_, NO_x_ and N_2_. The gas mixture entered a quartz glass reduction tube filled (from the bottom) with ~0.5 cm glass wool, ~35 cm copper and hold at 680 °C to reduce NO_x_ completely to N_2_ and again ~0.5 cm glass wool (all parts purchased from IVA Analysentechnik e.K., Meerbusch, Germany) parts. The reduction reactor additionally acts as oxygen scavenger. After gas drying in a magnesium perchlorate filled tube the remaining gases were separated on a packed stainless steel column CHNS-IRMS, 2 m (IVA Analysentechnik e.K., Meerbusch, Germany) and introduced *via* the split of the ConFloIV interface into the isotope ratio mass spectrometer. The oven temperature for the gas separation was set to 40 °C. EA-IRMS values were obtained by identical treatment and normalization according to Werner & Brand [28].

All isotope ratios were reported in the *δ*-notation as differences of the isotope ratio of the sample and isotope ratio of an international reference substance by Eq. 1.

1 $$ {\delta}^h\;{\mathrm{E}}_{s, ref}=\frac{R{\left({}^h\mathrm{E}/{}^l\mathrm{E}\right)}_s}{R{\left({}^h\mathrm{E}/{}^l\mathrm{E}\right)}_{ref}}-1 $$

where, *R*(^*h*^E/^*l*^E)_*ref*_ denotes the ratio of the heavy and light isotope (here ^13^C/^12^C as well as ^15^N/^14^N) in the reference material (VPDB and AIR-N_2_) and *R*(^*h*^E/^*l*^E)_*s*_ in the sample.

As reference materials for normalization of the laboratory working standard acetanilide to the international scale, (urea; *δ* ^13^C = -45.031 ± 0.17‰; δ^15^N = -0.85 ± 0.16‰), and IAEA 600 (caffeine; *δ* ^13^C = -27.77 ± 0.04‰; *δ* ^15^N = 0.91 ± 0.09‰) were used. As internal laboratory standard, acetanilide (AcAn) (pro analysi, Merck, Germany) was normalized by a two point calibration against the international standards.

### Data analyses and statistical evaluation

Stable isotope mean values in terms of *δ* ^13^C and *δ* ^15^N were stated with confidence intervals (*CI*) with $\overline{x}\kern0.5em \pm \kern0.5em  t\left(0.95;\kern0.5em  f= n-1\right)\times s/\sqrt{n}$, where *n* is ten (summer) or eight (autumn).

Values for isotopic discrimination *Δδ* ^*h*^E_*host* − *parasite*_ for carbon and nitrogen between parasite and host were expressed by Eq. 2,

2 $$ \varDelta {\delta}^h{\mathrm{E}}_{h ost- parasite}={\delta}^h{\mathrm{E}}_{h ost}-{\delta}^h{\mathrm{E}}_{parasite} $$

where *δ* ^*h*^ *E*_*parasite*_ is the *δ*-value of the parasite tissue and *δ* ^*h*^ *E*_*host*_ that of the host tissue.

Differences in ratios of stable isotopes between host tissues and parasites were tested using Wilcoxon matched pair test. Mann-Whitney *U*-test was applied to compare the stable isotope composition of samples collected in summer and autumn. Spearman’s rank correlation analyses were used in order to evaluate possible relationships between the composition of stable isotopes in fish tissues and parasites.

## Results

### Stable isotope composition in fish-parasite system

**Fig. 1:**
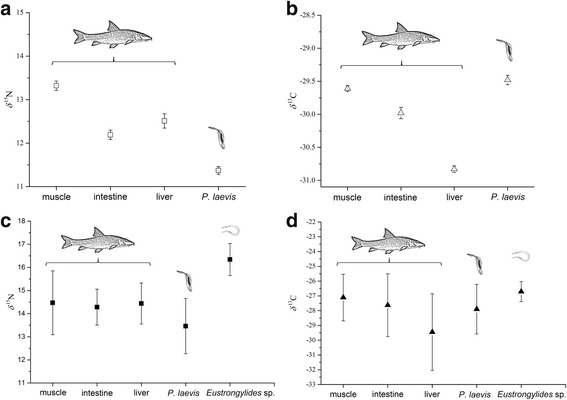
Composition of stable isotopes *δ* ^15^N and *δ* ^13^C in per mil in the selected host-parasite systems. **a**, **b** Summer. **c**, **d** Autumn (co-infection)

The isotope signatures in the selected host-parasite systems showed clear and contrasting patterns. In general, the acanthocephalans were depleted in *δ* ^15^N with respect to all host tissues in contrast to nematodes, which were enriched in *δ* ^15^N with respect to both acanthocephalans and host organs (Fig. 1, Table 2).

**Table 2:** Mean values ± CI with t (95%; f = *n*-1) × s/√*n* of *δ* ^13^C and *δ* ^15^N in per mil calculated for host tissues and parasites

	*δ* ^15^N ± CI		*δ* ^13^C ± CI	
	Summer (*n* = 10)	Autumn (*n* = 8)	Summer (*n* = 10)	Autumn (*n* = 8)
Muscle	13.3 ± 0.1	14.5 ± 1.4	-29.6 ± 0.0	-27.1 ± 1.6
Intestine	12.2 ± 0.1	14.3 ± 0.8	-30.0 ± 0.1	-27.6 ± 2.1
Liver	12.5 ± 0.2	14.4 ± 0.9	-30.8 ± 0.1	-29.5 ± 2.6
*P. laevis*	11.4 ± 0.1	13.5 ± 1.2	-29.5 ± 0.1	-27.9 ± 1.7
*Eustrongylides* sp.	–	16.3 ± 0.7	–	-26.7 ± 0.7

*Abbreviation*: *CI* confidence interval

The highest differences in nitrogen composition between *P. laevis* and its host were obtained for muscle tissues (Δ*δ* ^15^N = 2.0‰; Wilcoxon test, *Z =* 3.33, *P* < 0.001) followed by liver (Δ*δ* ^15^N = 1.1‰; Wilcoxon test, *Z =* 3.47, *P* < 0.001) and intestine (Δ*δ* ^15^N = 0.8‰; Wilcoxon test, *Z =* 3.52, *P* < 0.001) in summer, whereas the isotopic discrimination values of *Eustrongylides* sp. larvae remained similar for all host tissues and showed an isotopic discrimination for nitrogen between -1.9 and -2.1‰ (Wilcoxon test, Z ranged between 2.38–2.52; *P* < 0.05; see Table 3). Comparing the isotope discrimination values between the co-occurring parasite species, a clear enrichment of the heavier nitrogen isotope in nematodes with respect to acanthocephalans was observed (Δ *δ* ^15^N _nematodes *-* acanthocephalans*.*_ of 2.9‰; Wilcoxon test, *Z =* 2.52, *P* < 0.05; see Table 3, Fig. 1c).

**Table 3:** Δ *δ* ^13^C and Δ *δ* ^15^N values in per mil of isotope signatures calculated for parasites in relation to their host tissues and between both parasite species (Δ^h^E _*P. laevis - Eustrongylides* sp._)

Parasite	Muscle		Intestine		Liver		*P. laevis*	
	Δ*δ* ^15^N	Δ*δ* ^13^C	Δ*δ* ^15^N	*Δδ* ^13^C	Δ*δ* ^15^N	Δ*δ* ^13^C	Δ*δ* ^15^N	Δ*δ* ^13^C
*P. laevis* (summer)	2.0	-0.1	0.8	-0.5	1.1	-1.4	–	–
*P. laevis* (autumn)	1.0	0.8	0.8	0.3	1.0	-1.6	2.9	1.6
*Eustrongylides* sp.	-1.9	-0.4	-2.1	-0.9	-2.0	-2.8	-2.9	-1.2

The carbon isotope signatures in the host’s muscle and intestine tissues as well as in acanthocephalans were similar (Table 2, Fig. 1 b, d). The only differences were observed for the liver, which was depleted in *δ* ^13^C with about 1.5‰ (Wilcoxon test, *Z =* 2.59, *P* < 0.05) in comparison to *P. laevis* and other tissues, respectively. In contrast, the *Eustrongylides* sp. larvae were enriched in heavier carbon isotope in comparison to all host tissues with -0.4‰, -0.9‰ and -2.8‰, for muscle, intestine and liver tissues, respectively (see Table 3), whereas the only significant differences were observed for liver (Wilcoxon test, *Z =* 1.96, *P* < 0.05). Accordingly, the nematodes revealed higher carbon isotope discrimination values when compared with the acanthocephalans (Δ*δ* ^13^C = -1.2‰).

**Fig. 2:**
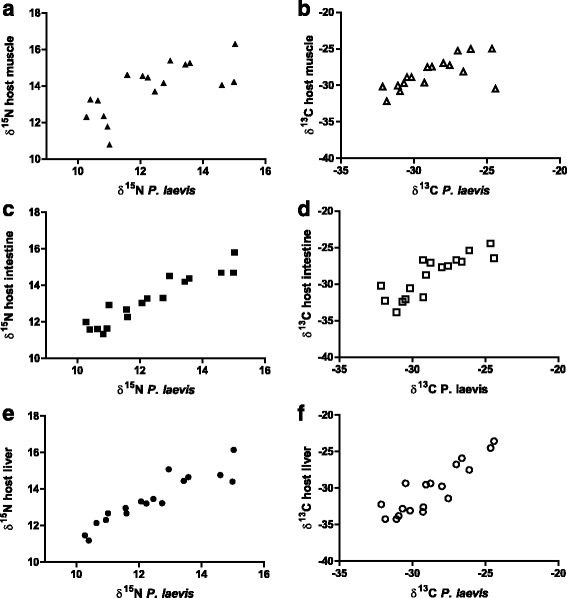
Relationship of *δ* ^15^N and *δ* ^13^C composition in per mil between different host tissues and the acanthocephalan *P. laevis.* **a** *δ* ^15^N muscle ↔ *P. laevis*. **b** *δ* ^13^C muscle ↔ *P. laevis*. **c** *δ* ^15^N intestine ↔ *P. laevis*. **d** *δ* ^13^C intestine ↔ *P. laevis*. **e** *δ* ^15^N liver ↔ *P. laevis*. **f** *δ* ^13^C liver ↔ *P. laevis*

**Fig. 3:**
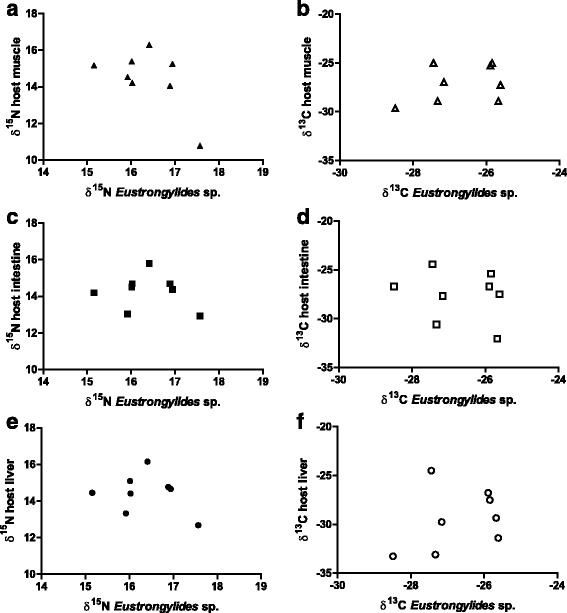
Relationship of *δ* ^15^N and *δ* ^13^C composition in per mil between different host tissues and the nematode *Eustrongylides* sp. **a** *δ* ^15^N muscle ↔ *Eustrongylides* sp. **b** *δ* ^13^C muscle ↔ *Eustrongylides* sp. **c** *δ* ^15^N intestine ↔ *Eustrongylides* sp. **d** *δ* ^13^C intestine ↔ *Eustrongylides* sp. **e** *δ* ^15^N liver ↔ *Eustrongylides* sp. **f** *δ* ^13^C liver ↔ *Eustrongylides* sp.

The correlation analyses of stable isotope composition in the parasites showed no significant relationship with the morphological parameters of fish. However, isotope signatures of acanthocephalans correlated significantly (*P* < 0.05) with those of host tissues (Fig. 2). Thus, the highest correlation coefficients were obtained for intestinal tissue (*δ* ^15^N: *r* = 0.96; *δ* ^13^C: *r* = 0.86) followed by liver (*δ* ^15^N: *r* = 0.94; *δ* ^13^C: *r* = 0.83) and muscle (*δ* ^15^N: *r* = 0.70; *δ* ^13^C: *r* = 0.67). The correlation analyses performed between nematodes and host tissues revealed no significant (*P* > 0.05) relationships (see Fig. 3).

### Seasonal patterns of stable isotope composition in fish-parasite system

The isotope signatures of the selected fish-parasite systems exhibited differences between summer and autumn when host tissues and acanthocephalans are observed. These can either be attributed to seasonal differences or may be a result from the co-infection which was only found in barbel sampled in autumn. In general, the fish and parasite samples collected in autumn exhibited higher nitrogen and carbon isotope discrimination values than those collected in summer (Mann-Whitney, *Z* ranged from -1.97 to -3.03; *P* < 0.05 for all host tissues and acanthocephalans; see also Table 2). However, the calculated isotopic discrimination of acanthocephalans with respect to host tissues showed a similar pattern in most cases. For both seasons, the highest differences were obtained for *P. laevis* with respect to host muscle followed by those of liver and intestine (see Table 3). The isotopic discrimination calculated for muscle tissue with respect to *P. laevis* in summer were approximately 2‰ in comparison to autumn with a difference of 1‰. The differences in isotope signatures calculated for the other organs remained similar (intestine Δ*δ* ^15^N = 0.8‰, Δ*δ* ^13^C = 0.8‰; liver Δ*δ* ^15^N = 1.0‰, Δ*δ* ^13^C = 1.1‰).

## Discussion

The results of the present study revealed different fractionation patterns of stable isotopes in the selected parasite species. In general, the nematodes were enriched by approximately 2‰ in the heavier nitrogen isotope with respect to their host in contrast to the acanthocephalans, which were depleted in the range between 1 and 2‰. Accordingly, the larval nematodes were on a higher trophic level with significant *δ* ^15^N enrichment compared with the host, which resembles a consumer-diet discrimination pattern [12]. In contrast, the acanthocephalans were on a lower trophic level than the fish host and the nematodes, respectively. The differences between the investigated parasite species could be attributed to differences in their taxonomic position, their feeding strategy, their developmental stage and their localization in the host.

Overall, the parasite’s taxonomy in combination with its way of nutrient uptake determines its trophic position with respect to the host. Although there are no data available regarding stable isotope composition of acanthocephalans, the results of the present study were in line with some other groups of parasites, which share a similar absorptive feeding strategy. For example, cestodes were reported to be depleted in *δ* ^15^N and *δ* ^13^C [14, 17, 20]. These findings were also opposed to a predator–prey relationship, that defines parasites as consumers, which feed on their hosts [29]. Cestodes as well as acanthocephalans take up nutrients through the body surface (tegument) and assimilate compounds, which were previously processed by the host. These metabolites (peptides, amino acids, carbohydrates mono-oligosaccharides) are depleted in heavier stable isotopes, as due to the kinetic isotope effect, lighter isotopes are favored in biochemical reactions [30]. Parasites, in general, face limitations in metabolism and energy utilization under anaerobic conditions and various macromolecules have to be assimilated *via* the tegument [31]. It is also known that endoparasites are not able to synthetize several complex molecules such as purine nucleotides, fatty acids, sterols, and some amino acids *de novo*. The only source for these molecules remains the host’s metabolism [31, 32]. Ammonia to be excreted by the host, which is enriched in the lighter nitrogen isotope, can also be taken up and metabolized by endoparasites [33]. Bile fluids produced by the host, which become available for intestinal parasites *via* the entero-hepatic cycle, were found to play a significant role for the growth and reproduction of acanthocephalans in the definitive host [34–37] and for the uptake of metals [38, 39]. Additionally, selective membrane transport mechanisms, which might favor the uptake of isotopically lighter molecules, could improve the parasite’s metabolism efficiency and energy utilization under anaerobic conditions. The high correlation coefficients obtained between isotopic values of host tissues (e.g. liver and intestine) and acanthocephalans gives further evidence for a direct uptake of host metabolites. Accordingly, the uptake of host metabolites through the tegument could explain lower isotope discrimination values of parasitic taxa exhibiting an assimilation strategy, such as the acanthocephalans and the cestodes.

The stable isotope signatures of the fish-nematode system in our study showed a consumer-diet pattern. In contrast to acanthocephalans, nematodes possess a completely developed gastrointestinal tract. Larval eustrongylids were found mainly free in the body cavity of fish, where they feed actively on host tissues and body fluids. Therefore, similar to consumers, they were enriched in heavier nitrogen isotopes in relation to host tissues. Such patterns were also reported for adult nematodes from their definitive host, as they exhibit a similar feeding strategy [16, 40, 41]. The larval *Eustrongylides* sp. in barbel were characterized as fourth-stage larvae (L4), which exhibit close morphological and feeding similarities to the adults. Latter were found to parasitize the outside wall of the proventriculus and other tissues of fish-eating birds, where it might cause severe histological damage as observed for the larval stages in fish [24, 42, 43]. The lacking relationship between the stable isotope composition of the nematodes and host’s tissues might be attributed to their life span and behavior in the second intermediate host. In contrast to fast growing acanthocephalans larval nematodes can live in the host for many months over different seasons before being transmitted to a definitive host. However, after molting into fourth-stage larva (L4) in the fish host they exhibit much lower metabolic rates than the actively growing and reproducing acanthocephalans. Therefore, changes in host food composition and feeding activity and an associated isotopic shift have most probably no such strong influence on the composition of stable isotopes in nematodes as observed for the acanthocephalans.

The observed seasonality in infection dynamics [23] supports the above-mentioned aspects on nutrient uptake and utilization of acanthocephalans. The seasonal shifts in isotope discrimination values of acanthocephalans followed exactly those of the metabolically active tissues of the host. The differences between fishes collected in summer and autumn can be attributed to changes in fish activity [44]. The barbel exhibits a higher feeding activity in autumn [45], which was suggested to be a compensation mechanism for the dormancy phase during winter months at temperatures around or below 4 °C [46]. This, combined with changes in food composition, could explain differences in the isotopic composition of the host tissues, which might similarly also have affected those of acanthocephalans, as they assimilate nutrients processed by the host.

## Conclusions

The results of the present study unravel differences concerning the trophic interaction between different parasite taxa and their hosts. We provided first data for acanthocephalans, which supported the tendency already described for other taxa with similar nutrition strategy like cestodes. Actively feeding taxa such as larval *Eustrongylides* sp., act like predators as it can be seen from their isotope discrimination values. However, future research on additional host-parasite systems and especially on acanthocephalans is needed in order to corroborate these conclusions. Future research should also address the entire life-cycle of heteroxenous parasites, taking into account all larval stages and involved hosts.
